# An improved multiply robust estimator for the average treatment effect

**DOI:** 10.1186/s12874-023-02056-7

**Published:** 2023-10-11

**Authors:** Ce Wang, Kecheng Wei, Chen Huang, Yongfu Yu, Guoyou Qin

**Affiliations:** 1 Department of Biostatistics, Key Laboratory for Health Technology Assessment, National Commission of Health, Key Laboratory of Public Health Safety of Ministry of Education, School of Public Health, Fudan University, Shanghai, China; 2 Shanghai Institute of Infectious Disease and Biosecurity, Shanghai, China

**Keywords:** Average treatment effect, Empirical likelihood, Multiply robust, Nonparametric model, Parametric model

## Abstract

**Background:**

In observational studies, double robust or multiply robust (MR) approaches provide more protection from model misspecification than the inverse probability weighting and g-computation for estimating the average treatment effect (ATE). However, the approaches are based on parametric models, leading to biased estimates when all models are incorrectly specified. Nonparametric methods, such as machine learning or nonparametric double robust approaches, are robust to model misspecification, but the efficiency of nonparametric methods is low.

**Method:**

In the study, we proposed an improved MR method combining parametric and nonparametric models based on the previous MR method (Han, JASA 109(507):1159-73, 2014) to improve the robustness to model misspecification and the efficiency. We performed comprehensive simulations to evaluate the performance of the proposed method.

**Results:**

Our simulation study showed that the MR estimators with only outcome regression (OR) models, where one of the models was a nonparametric model, were the most recommended because of the robustness to model misspecification and the lowest root mean square error (RMSE) when including a correct parametric OR model. And the performance of the recommended estimators was comparative, even if all parametric models were misspecified. As an application, the proposed method was used to estimate the effect of social activity on depression levels in the China Health and Retirement Longitudinal Study dataset.

**Conclusions:**

The proposed estimator with nonparametric and parametric models is more robust to model misspecification.

**Supplementary Information:**

The online version contains supplementary material available at 10.1186/s12874-023-02056-7.

## Background

The primary goal of much-applied research is to estimate the causal effect of specific treatment (exposure or intervention) on the outcome. In randomized controlled trials, where treatments are randomly assigned to participants, the average treatment effect (ATE) can be estimated by directly comparing outcomes between treatment and control groups [1]. In observational studies, however, there are usually unbalanced covariates between treatment and control groups due to the non-randomized treatment assignment. As a result, a direct comparison of outcomes between treatment and control groups may lead to a biased estimation of the ATE [2].

The inverse probability weighting (IPW) with a propensity score (PS) model and the direct confounding adjustment (known as g-computation) with an outcome regression (OR) model are the general approaches to handling confounding bias [1, 3, 4]; Compared to IPW and g-computation methods, doubly robust approaches provide double protection from model misspecification [5–7]; but, the doubly robust approach does not offer sufficient protection for estimating ATE in practice, as they allow only one PS model and one OR model. Recently, multiply robust (MR) approaches, increasing the likelihood of including the correct model, are proposed for estimating ATE or a population mean with missing values [8–11].And, the previous MR approach [9] is robust against extreme values of the fitted receiving treatment probability. However, the previous MR approach [9] only considering parametric models may lead to a biased estimation when the included parametric models are all incorrectly specified.

In addition, there is growing interest in developing nonparametric methods for estimating ATE to protect against model misspecification. Machine learning, a general term for a diverse number of nonparametric algorithms, is particularly useful for classification and prediction and is used to estimate the ATE [12–17]. However, the root mean square error (RMSE) of machine learning seems to be higher than that of the correct parametric model may due to incorrect hyperparameter settings [18–20]. Nonparametric double robust methods based on the kernel smoothing approach [21, 22] or targeted minimum loss [23] have also been proposed to estimate the ATE. Yet, the efficiency of these estimators is not high because of slow convergence rates.

In this study, based on the previous MR approach [9], we proposed an improved MR approach considering both parametric and nonparametric models to improve the robustness to model misspecification. Our simulation study showed that the proposed MR approach is more robust to model misspecification than previous MR approach; and the MR estimators with only OR models, where one of the models was a nonparametric model, were the best among all MR estimators for the following two reasons. First, the MR estimators were robust to model misspecification, and had the lowest root mean square errors (RMSEs) when including a correct parametric OR model. Second, the performance of the best estimators was comparative even if all parametric models were misspecified.

## Method

### Notation and assumptions

Let ${{\varvec{X}}}_{i}$ be a $p$-dimensional vector of covariates, ${Y}_{i}$ be the observed outcome, and ${Z}_{i}$ be the treatment status taking value 1 if treated or 0 if untreated. Let (${Y}^{1}, {Y}^{0}$) be the two potential outcomes in the treatment and control groups, respectively, and the ATE is defined as

$$\Delta =E\left({Y}^{1}\right)-E\left({Y}^{0}\right)$$

And to draw a correct causal inference in the study, exchangeability, consistency, and positivity assumptions hold [24].

### Previous multiply robust method

The previous MR approach proposed by Han [9] provides multiple protection to the model misspecification. Specifically, specifying two sets of parametric models, $\mathcal{P}=\left\{{\pi }^{l}\left({\varvec{X}}\right),l=1, 2, 3,\dots ,L\right\}$ for propensity score and $\mathcal{M}=\left\{{m}_{z}^{k}\left({\varvec{X}}\right),k=\mathrm{1,2},3,\dots ,K\right\}$ for outcome, where ${m}_{z}^{k}\left({\varvec{X}}\right)={m}^{k}\left({\varvec{X}},Z\right)$. Without loss of generality, let ${\mathbb{I}}=1,\dots ,{n}_{1}$ and ${\mathbb{J}}=1,\dots ,{n}_{0}$ be the indexes for treated and untreated subjects, respectively. Let ${n}_{1}$ and ${n}_{0}=n-{n}_{1}$ represent the size of treatment and control groups, respectively.

To recover the treated population average from subjects in the treatment group, the empirical likelihood weights ${w}_{i}\left(i\in {\mathbb{I}}\right)$ for the outcome ${Y}_{i}\left(i\in {\mathbb{I}}\right)$ in the treatment group are estimated by maximizing $\prod_{i\in {\mathbb{I}}}{w}_{i}$ subject to the following constraints:

$${w}_{i}\ge 0 (i\in {\mathbb{I}})$$

$$\sum_{i\in {\mathbb{I}}}{w}_{i}=1$$

$$\sum_{i\in {\mathbb{I}}}{w}_{i}{\widehat{\pi }}^{l}\left({{\varvec{X}}}_{i}\right)={\widehat{\theta }}_{1}^{l}(l=1, 2, 3,\dots ,L)$$

$$\sum_{i\in {\mathbb{I}}}{w}_{i}{\widehat{m}}_{1}^{k}\left({{\varvec{X}}}_{i}\right)={\widehat{\eta }}_{1}^{k}(k=1, 2, 3,\dots ,K)$$

where ${\widehat{\theta }}_{1}^{l}={n}^{-1}{\sum }_{i=1}^{n}{\pi }^{l}\left({{\varvec{X}}}_{i}\right)$ and ${\widehat{\eta }}_{1}^{k}={n}^{-1}{\sum }_{i=1}^{n}{m}_{1}^{k}\left({{\varvec{X}}}_{i}\right)$. By symmetry, the weights ${w}_{j}\left(j\in {\mathbb{J}}\right)$ for the control group are given by maximizing $\prod_{j\in {\mathbb{J}}}{w}_{j}$ according to the following constraints:

$${w}_{j}\ge 0 (j\in {\mathbb{J}})$$

$$\sum_{j\in {\mathbb{J}}}{w}_{j}=1$$

$$\sum_{j\in {\mathbb{J}}}{w}_{j}({1-\widehat{\pi }}^{l}\left({{\varvec{X}}}_{j}\right))={\widehat{\theta }}_{0}^{l}(l=1, 2, 3,\dots ,L)$$

$$\sum_{j\in {\mathbb{J}}}{w}_{j}{\widehat{m}}_{0}^{k}\left({{\varvec{X}}}_{j}\right)={\widehat{\eta }}_{0}^{k}(k=1, 2, 3,\dots ,K)$$

where ${\widehat{\theta }}_{0}^{l}={n}^{-1}{\sum }_{i=1}^{n}(1-{\pi }^{l}\left({{\varvec{X}}}_{i}\right))$ and ${\widehat{\eta }}_{0}^{k}={n}^{-1}{\sum }_{i=1}^{n}{m}_{0}^{k}\left({{\varvec{X}}}_{i}\right)$. The ${w}_{i}$ and ${w}_{j}$ can be given with Lagrange multiplier method as follows:

$${\widehat w}_i=\frac1{n_1}\frac1{1+\widehat p_1^T{\widehat g}_1\left(X_i\right)}\left(i\in\mathbb{I}\right)$$

$${\widehat w}_j=\frac1{n_0}\frac1{1+\widehat p_0^T{\widehat g}_0\left(X_j\right)}\left(j\in\mathbb{J}\right)$$

where

$${\widehat{{\varvec{g}}}}_{1}{\left({\varvec{X}}\right)}^{T}=\left\{{\widehat{\pi }}^{1}\left({\varvec{X}}\right)-{\widehat{\theta }}_{1}^{1},\dots ,{\widehat{\pi }}^{L}\left({\varvec{X}}\right)-{\widehat{\theta }}_{1}^{L}, {\widehat{m}}_{1}^{1}\left({\varvec{X}}\right)-{\widehat{\eta }}_{1}^{1},\dots ,{\widehat{m}}_{1}^{K}\left({\varvec{X}}\right)-{\widehat{\eta }}_{1}^{K}\right\}$$

$${\widehat{{\varvec{g}}}}_{0}{\left({\varvec{X}}\right)}^{T}=\left\{{(1-\widehat{\pi }}^{1}\left({\varvec{X}}\right)\right)-{\widehat{\theta }}_{0}^{1},\dots ,(1-{\widehat{\pi }}^{L}\left({\varvec{X}}\right))-{\widehat{\theta }}_{0}^{L}, {\widehat{m}}_{0}^{1}\left({\varvec{X}}\right)-{\widehat{\eta }}_{0}^{1},\dots ,{\widehat{m}}_{0}^{K}\left({\varvec{X}}\right)-{\widehat{\eta }}_{0}^{K}\}$$

${\widehat{{\varvec{\rho}}}}_{1}^{T}$ and ${\widehat{{\varvec{\rho}}}}_{0}^{T}$ are the $(J+K$)-dimensional Lagrange multipliers solving

$$\frac1{n_1}\sum\limits_{i\in\mathbb{I}}\frac{{\widehat{{\varvec{g}}}}_1\left(\boldsymbol{X}_i\right)}{1+{\widehat{{\varvec{\rho}}}}_1^T{\widehat{{\varvec{g}}}}_1\left(\boldsymbol{X}_i\right)}=0$$

$$\frac1{n_0}\sum\limits_{j\in\mathbb{I}}\frac{{\widehat{{\varvec{g}}}}_0\left(\boldsymbol{X}_j\right)}{1+{\widehat{{\varvec{\rho}}}}_0^T{\widehat{{\varvec{g}}}}_0\left(\boldsymbol{X}_j\right)}=0$$

${\widehat{{\varvec{\rho}}}}_{1}^{T}$ and ${\widehat{{\varvec{\rho}}}}_{0}^{T}$ must satisfy $1+{\widehat{\rho }}_{1}^{T}{\widehat{g}}_{1}\left({{\varvec{X}}}_{i}\right)>0$ and $1+{\widehat{\rho }}_{0}^{T}{\widehat{g}}_{0}\left({{\varvec{X}}}_{j}\right)>0$ due to the non-negativity of ${w}_{i}$ and ${w}_{j}$, respectively. The estimation of ${\widehat{w}}_{i}$ and ${\widehat{w}}_{j}$ can be solved by the Newton–Raphson algorithm [9].

In summary, the ATE estimated by MR method [9] is defined as

$${\widehat{\Delta }}_{mr(Han)}=\sum_{i\in {\mathbb{I}}}{\widehat{w}}_{i}{Y}_{i}-\sum_{j\in {\mathbb{J}}}{\widehat{w}}_{j}{Y}_{j}$$

### Proposed multiply robust method

The previous MR method [9] allows multiple parametric models, increasing the likelihood of including the correct model; However, there is still a biased estimates of ATE when all parametric models are misspecified. Based on the previous MR method, the proposed MR method allows multiple parametric models, and also includes a nonparametric PS model and a nonparametric OR model.

We select the neural network (NNET) as nonparametric models in the proposed MR method. NNET, one machine learning algorithm, has been used to estimate the ATE [15, 16]. We specified three-layer (input layer, one hidden layer, output layer) NNET, which may be practical [12], and the hidden layer consists of 4 nodes. We performed the NNET using the *nnet* R package with default parameters. Finally, the proposed MR method added a NNET-based outcome regression model (NN-OR) and a NNET-based propensity score model (NN-PS) in base of previous MR method.

Similar to the previous MR method [9], we specify two sets of models, $\mathcal{P}=\left\{{\pi }^{l}\left({\varvec{X}}\right),l=1, 2, 3,\dots ,L,L+1\right\}$ for propensity score and $\mathcal{M}=\left\{{m}_{z}^{k}\left({\varvec{X}}\right),k=\mathrm{1,2},3,\dots ,K,K+1\right\}$ for outcome. Assume ${\pi }^{L+1}\left({\varvec{X}}\right)$ and ${m}_{z}^{K+1}({\varvec{X}})$ are the NN-PS and NN-OR models, respectively, and the other are parametric models. The empirical likelihood weights ${w}_{i}\left(i\in {\mathbb{I}}\right)$ for the outcome ${Y}_{i}\left(i\in {\mathbb{I}}\right)$ in the treatment group are estimated by maximizing $\prod_{i\in {\mathbb{I}}}{w}_{i}$ subject to the following constraints:

$${w}_{i}\ge 0 (i\in {\mathbb{I}})$$

$$\sum_{i\in {\mathbb{I}}}{w}_{i}=1$$

$$\sum_{i\in {\mathbb{I}}}{w}_{i}{\widehat{\pi }}^{l}\left({{\varvec{X}}}_{i}\right)={\widehat{\theta }}_{1}^{l}(l=1, 2, 3,\dots ,L,L+1)$$

$$\sum_{i\in {\mathbb{I}}}{w}_{i}{\widehat{m}}_{1}^{k}\left({{\varvec{X}}}_{i}\right)={\widehat{\eta }}_{1}^{k}(k=1, 2, 3,\dots ,K,K+1)$$

where ${\widehat{\theta }}_{1}^{l}={n}^{-1}{\sum }_{i=1}^{n}{\pi }^{l}\left({{\varvec{X}}}_{{\varvec{i}}}\right)$ and ${\widehat{\eta }}_{1}^{k}={n}^{-1}{\sum }_{i=1}^{n}{m}_{1}^{k}\left({{\varvec{X}}}_{i}\right)$. By symmetry, the weights ${w}_{j}\left(j\in {\mathbb{J}}\right)$ for the control group are given by maximizing $\prod_{j\in {\mathbb{J}}}{w}_{j}$ according to the following constraints:

$${w}_{j}\ge 0 (j\in {\mathbb{J}})$$

$$\sum_{j\in {\mathbb{J}}}{w}_{j}=1$$

$$\sum_{j\in {\mathbb{J}}}{w}_{j}({1-\widehat{\pi }}^{l}\left({{\varvec{X}}}_{j}\right))={\widehat{\theta }}_{0}^{l}(l=1, 2, 3,\dots ,L,L+1)$$

$$\sum_{j\in {\mathbb{J}}}{w}_{j}{\widehat{m}}_{0}^{k}\left({{\varvec{X}}}_{j}\right)={\widehat{\eta }}_{0}^{k}(k=1, 2, 3,\dots ,K,K+1)$$

where ${\widehat{\theta }}_{0}^{l}={n}^{-1}{\sum }_{i=1}^{n}(1-{\pi }^{l}\left({{\varvec{X}}}_{i}\right))$ and ${\widehat{\eta }}_{0}^{k}={n}^{-1}{\sum }_{i=1}^{n}{m}_{0}^{k}\left({{\varvec{X}}}_{i}\right)$. The ${w}_{i}$ and ${w}_{j}$ can be given with Lagrange multiplier method as follows:

$${\widehat{w}}_{i}=\frac{1}{{n}_{1}}\frac{1}{1+{\widehat{{\varvec{\rho}}}}_{1}^{T}{\widehat{{\varvec{g}}}}_{1}\left({{\varvec{X}}}_{i}\right)} \left(i\in {\mathbb{I}}\right)$$

$${\widehat{w}}_{j}=\frac{1}{{n}_{0}}\frac{1}{1+{\widehat{{\varvec{\rho}}}}_{0}^{T}{\widehat{{\varvec{g}}}}_{0}\left({{\varvec{X}}}_{j}\right)} \left(j\in {\mathbb{J}}\right)$$

where

$${\widehat{{\varvec{g}}}}_{1}{\left({\varvec{X}}\right)}^{T}=\left\{{\widehat{\pi }}^{1}\left({\varvec{X}}\right)-{\widehat{\theta }}_{1}^{1},\dots ,{\widehat{\pi }}^{L}\left({\varvec{X}}\right)-{\widehat{\theta }}_{1}^{L},{\widehat{\pi }}^{L+1}\left({\varvec{X}}\right)-{\widehat{\theta }}_{1}^{L+1}, {\widehat{m}}_{1}^{1}\left({\varvec{X}}\right)-{\widehat{\eta }}_{1}^{1},\dots ,{\widehat{m}}_{1}^{K}\left({\varvec{X}}\right)-{\widehat{\eta }}_{1}^{K},{\widehat{m}}_{1}^{K+1}\left({\varvec{X}}\right)-{\widehat{\eta }}_{1}^{K+1}\right\}$$

$${\widehat{{\varvec{g}}}}_{0}{\left({\varvec{X}}\right)}^{T}=\left\{{(1-\widehat{\pi }}^{1}\left({\varvec{X}}\right)\right)-{\widehat{\theta }}_{0}^{1},\dots ,\left(1-{\widehat{\pi }}^{L}\left({\varvec{X}}\right)\right)-{\widehat{\theta }}_{0}^{L}, \left(1-{\widehat{\pi }}^{L+1}\left({\varvec{X}}\right)\right)-{\widehat{\theta }}_{0}^{L+1}, {\widehat{m}}_{0}^{1}\left({\varvec{X}}\right)-{\widehat{\eta }}_{0}^{1},\dots ,{\widehat{m}}_{0}^{K}\left({\varvec{X}}\right)-{\widehat{\eta }}_{0}^{K},{\widehat{m}}_{0}^{K+1}\left({\varvec{X}}\right)-{\widehat{\eta }}_{0}^{K+1}\}$$

${\widehat{{\varvec{\rho}}}}_{1}^{T}$ and ${\widehat{{\varvec{\rho}}}}_{0}^{T}$ are the $(J+K+2$)-dimensional Lagrange multipliers solving

$$\frac1{n_1}\sum\limits_{i\in\mathbb{I}}\frac{{\widehat{{\varvec{g}}}}_1\left(\boldsymbol{X}_i\right)}{1+{\widehat{{\varvec{\rho}}}}_1^T{\widehat{{\varvec{g}}}}_1\left(\boldsymbol{X}_i\right)}=0$$

$$\frac1{n_0}\sum\limits_{i\in\mathbb{J}}\frac{{\widehat{{\varvec{g}}}}_0\left(\boldsymbol{X}_j\right)}{1+{\widehat{{\varvec{\rho}}}}_0^T{\widehat{{\varvec{g}}}}_0\left(\boldsymbol{X}_j\right)}=0$$

${\widehat{{\varvec{\rho}}}}_{1}^{T}$ and ${\widehat{{\varvec{\rho}}}}_{0}^{T}$ must satisfy $1+{\widehat{{\varvec{\rho}}}}_{1}^{{\varvec{T}}}{\widehat{{\varvec{g}}}}_{1}\left({{\varvec{X}}}_{i}\right)>0$ and $1+{\widehat{{\varvec{\rho}}}}_{0}^{T}{\widehat{{\varvec{g}}}}_{0}\left({{\varvec{X}}}_{j}\right)>0$ due to the non-negativity of ${w}_{i}$ and ${w}_{j}$, respectively. The estimation of ${\widehat{w}}_{i}$ and ${\widehat{w}}_{j}$ can be solved by the Newton–Raphson algorithm [9].

The ATE estimated by the proposed method is defined as

$${\widehat{\Delta }}_{mr}=\sum_{i\in {\mathbb{I}}}{\widehat{w}}_{i}{Y}_{i}-\sum_{j\in {\mathbb{J}}}{\widehat{w}}_{j}{Y}_{j}$$

### Bootstrap confidence interval

The confidence interval of the estimators $\Delta$ could be obtained by the bootstrap method, where $\Delta$ maybe IPW, OR, or MR estimator. Specifically, $n$ individuals first are resampled with replacement from the original data for $B$ times to obtain $B$ bootstrap sample, where $B$ is the pre-specified number. For $b=1,\dots ,B$, let ${\widehat{\Delta }}^{b}$ be the estimates of the estimator from the $b$-th bootstrap sample. Then the bootstrap variance estimator for ${\widehat{\Delta }}^{b}$ is given by

$$\widehat{var}\left({\widehat{\Delta }}^{b}\right)= \frac{1}{B-1}\sum_{b=1}^{B}({\widehat{\Delta }}^{b}-\frac{1}{B}\sum_{b=1}^{B}{\widehat{\Delta }}^{b}{)}^{2}$$

A normality-based 95% confidence interval for $\Delta$ is ${\widehat{\Delta }}^{b}\pm 1.96\sqrt{\widehat{var}\left({\widehat{\Delta }}^{b}\right)}$

### Simulation study

#### Simulation design

We conducted comprehensive simulation studies to evaluate the performance of the proposed MR method. Ten covariates ${X}_{1}-{X}_{10}$ were simulated from the standard normal distribution, where $\mathrm{corr}\left({X}_{1},{X}_{5}\right)=\mathrm{corr}\left({X}_{4},{X}_{9}\right)=0.9$ and $\mathrm{corr}\left({X}_{2},{X}_{6}\right)=\mathrm{corr}\left({X}_{3},{X}_{8}\right)=0.2$. The treatment indicator was generated from a Bernoulli distribution with a PS model as followed

$$logit[P(Z=1|{\varvec{X}})]=0.8*{X}_{1}-0.25*{X}_{2} + 0.6*{X}_{3}-0.4*{X}_{4}-0.8*{X}_{5}-0.5*{X}_{6}+ 0.7*{X}_{7}$$,

which produced a treatment prevalence of approximately 50%. The outcome was simulated from

$$Y=-3.85 + 0.3*{X}_{1}-0.36*{X}_{2}-0.73*{X}_{3}-0.2*{X}_{4} + 0.71*{X}_{8} + 0.19*{X}_{9} + 0.26*{X}_{10} + 0.3*{X}_{1}^{2}-0.36*{X}_{2}^{2} + Z + \varepsilon$$

where $\varepsilon \sim N(\mathrm{0,1})$. The true value of ATE is 1 in the simulation.

In the simulation, we specified three models, including a NN-PS model and two parametric models

$${\mathbb{A}}=\left\{\begin{array}{c}{\pi }^{1}(X)={f}_{1}({X}_{1},{X}_{2},{X}_{3},{{X}_{4},X}_{5},{X}_{6},{X}_{7})\\ {\pi }^{2}(X)={f}_{2}\left({X}_{1},{X}_{2},{X}_{3}{,{X}_{4},X}_{5},{X}_{6},{X}_{7}\right)\\ {\pi }^{3}(X)={f}_{3}\left({X}_{1}^{2},{X}_{2}^{2},{X}_{3}^{2},{X}_{4}^{2},{X}_{5}^{2},{X}_{6}^{2},{X}_{7}^{2}\right)\end{array}\right\}$$

for propensity score. Let ${\pi }^{1}({\varvec{X}})$ be the PS of NN-PS model, and ${\pi }^{2}({\varvec{X}})$ and ${\pi }^{3}\left({\varvec{X}}\right)$ be the PS of logistic models. And we also specified three models, including a OR-NNET model and two parametric models

$${\mathbb{B}}=\left\{\begin{array}{c}{m}^{1}(X,Z)={h}_{1}({X}_{1},{X}_{2},{X}_{3},{{X}_{4},X}_{8},{X}_{9},{X}_{10},Z)\\ \begin{array}{c}{m}^{2}\left({\varvec{X}},Z\right)={h}_{2}\left({X}_{1},{X}_{2},{X}_{3}{,X}_{8},{X}_{9},{X}_{10},{X}_{1}^{2},{X}_{2}^{2},Z\right)\\ {m}^{3}\left({\varvec{X}},Z\right)={h}_{3}\left({X}_{1}{X}_{2},{X}_{3}{X}_{4},{X}_{8}{X}_{9},{X}_{1}{X}_{8},{X}_{2}{X}_{9},{X}_{3}{X}_{10},Z\right)\end{array}\end{array}\right\}$$

for outcome regression. Let ${m}^{1}({\varvec{X}},Z)$ be the outcome of NN-OR model, and ${m}^{2}({\varvec{X}},Z)$ and ${m}^{3}\left({\varvec{X}},Z\right)$ be the outcome of linear regression models. According to the data-generating process, ${\pi }^{2}\left({\varvec{X}}\right)$ and ${m}^{2}\left({\varvec{X}},{\varvec{Z}}\right)$ were correctly specified. In order to distinguish these estimation methods, three IPW estimators were defined as “IPW.model1”, “IPW.model2” and “IPW.model3”, where “IPW.model1” refer to the IPW estimator with a NN-PS model; three OR estimators were defined as “OR.model1”, “OR.model2” and “OR.model3”, where “OR.model1” refer to the OR estimator with NN-OR model. For the MR estimators, each estimator is denoted as “MR000000” where each digit of the six numbers, from left to right, indicates if ${\pi }^{1}\left({\varvec{X}}\right)$, ${\pi }^{2}\left({\varvec{X}}\right)$, ${\pi }^{3}\left({\varvec{X}}\right)$,${m}^{1}\left({\varvec{X}},Z\right), {m}^{2}\left({\varvec{X}},Z\right)$ or ${m}^{3}\left({\varvec{X}},Z\right)$ is included in the estimator (“1” means yes, and “0” means no).

And we studied the performance of MR estimators when all parametric models were misspecified as follows

$${\mathbb{A}}=\left\{\begin{array}{c}{\pi }^{1}(X)={f}_{1}({X}_{1},{X}_{2},{X}_{3},{{X}_{4},X}_{5},{X}_{6},{X}_{7})\\ {\pi }^{2}\left({\varvec{X}}\right)={f}_{2}\left({X}_{1},{X}_{2}{,X}_{5}\right)\\ {\pi }^{3}(X)={f}_{3}\left({X}_{1}^{2},{X}_{2}^{2},{X}_{3}^{2},{X}_{4}^{2},{X}_{5}^{2},{X}_{6}^{2},{X}_{7}^{2}\right)\end{array}\right\}$$

for propensity score

$${\mathbb{B}}=\left\{\begin{array}{c}{m}^{1}(X,Z)={h}_{1}({X}_{1},{X}_{2},{X}_{3},{{X}_{4},X}_{8},{X}_{9},{X}_{10},Z)\\ \begin{array}{c}{m}^{2}\left({\varvec{X}},Z\right)={h}_{2}\left({X}_{1},{X}_{2},{X}_{10},Z\right)\\ {m}^{3}\left({\varvec{X}},Z\right)={h}_{3}\left({X}_{1}{X}_{2},{X}_{3}{X}_{4},{X}_{8}{X}_{9},{X}_{1}{X}_{8},{X}_{2}{X}_{9},{X}_{3}{X}_{10},Z\right)\end{array}\end{array}\right\}$$

for outcome regression.

In addition, which factors related to the treatment and outcome were unknown in practice; hence, we also explored the performance of MR estimators when including NNET models with all covariates in situations where parametric models included correct models or did not include any correct model.

In the simulation, we calculated the mean relative bias, RMSE, and coverage rate to assess the performance of the proposed MR method. All results were based on 1000 simulation replications, and the sample sizes $n=200, 500 \mathrm{\ and}\ 2000$.

### Simulation results

Table 1 and Figure S1 showed the simulation results of estimating ATE where the proposed MR method included the correct parametric models. And Table 2 and Figure S2 showed the simulation results when there were no correct parametric models in MR method. We could get a few conclusions from the simulation studies.

**Table 1:** Simulation results with different sample sizes = 200, 500 or 2000 in the situation where the parametric models included the correct models and the neural network model included true covariates

Estimator	*N* = 200			*N* = 500			*N* = 2000		
	Bias (%)	RMSE	CR (%)	Bias (%)	RMSE	CR (%)	Bias (%)	RMSE	CR (%)
IPW.model1	6.356	0.624	98.2	5.464	0.273	99.5	2.539	0.115	97.2
IPW.model2	7.234	0.472	98.8	1.497	0.306	96.2	0.702	0.166	95.5
IPW.model3	67.238	0.708	27.0	66.532	0.680	0.5	67.402	0.677	0
OR.model1	-0.983	0.197	97.6	-1.139	0.112	98.3	0.484	0.054	98.1
OR.model2	0.713	0.157	94.4	-0.544	0.095	95.1	0.245	0.046	95.6
OR.model3	67.206	0.714	20.3	66.563	0.683	0.7	67.402	0.678	0
MR100000	6.289	0.372	98.1	4.006	0.210	97.4	1.397	0.100	95.5
MR010000	3.434	0.292	93.7	1.196	0.194	92.3	0.604	0.100	93.4
MR001000	67.323	0.708	15.9	66.650	0.681	0.4	67.405	0.677	0
MR000100	0.421	0.192	96.2	-0.975	0.112	97.6	0.455	0.055	97.8
MR000010	0.737	0.159	93.7	-0.441	0.097	95.2	0.253	0.046	95.3
MR000001	67.395	0.715	19.9	66.618	0.683	0.7	67.425	0.678	0
MR100100	1.217	0.274	97.4	-0.427	0.154	98.0	0.085	0.075	95.8
MR010010	1.053	0.197	94.3	-0.201	0.127	93.2	0.131	0.061	93.7
MR100010	1.058	0.244	97.0	0.077	0.135	97.3	0.206	0.061	93.8
MR010100	0.860	0.241	95.3	-0.223	0.147	95.1	0.048	0.075	95.9
MR110000	4.005	0.366	98.1	2.045	0.204	97.2	1.331	0.100	95.2
MR000110	-2.888	0.169	95.1	-1.898	0.099	96.1	-0.039	0.046	96.0
MR000101	0.887	0.194	95.9	-0.722	0.111	97.3	0.498	0.055	97.8
MR000011	1.155	0.159	94.2	-0.212	0.098	94.9	0.307	0.046	95.5
MR001100	0.484	0.181	97.8	-0.834	0.106	97.3	0.542	0.054	96.9
MR101000	5.245	0.389	98.5	4.602	0.219	97.9	1.414	0.098	94.6
MR001001	66.695	0.703	18.8	66.293	0.678	0.5	67.372	0.677	0
MR111000	1.872	0.374	98.5	2.412	0.212	97.7	1.324	0.098	95.1
MR000111	-2.446	0.168	94.9	-1.639	0.100	95.8	0.011	0.047	96.0
MR001101	1.076	0.275	97.0	-0.302	0.154	98.3	0.172	0.074	96.1
MR110100	1.500	0.270	96.9	-0.513	0.156	97.6	0.100	0.076	95.2
MR100110	0.677	0.247	97.1	-0.167	0.136	97.3	0.220	0.061	94.6
MR101101	0.215	0.269	97.6	-0.354	0.151	98.1	0.242	0.071	95.8
MR110110	1.180	0.244	96.8	-0.225	0.137	96.7	0.225	0.062	94.0
MR011011	1.383	0.204	94.5	0.060	0.131	94.5	0.209	0.062	94.2
MR111011	0.871	0.256	96.9	-0.110	0.139	97.5	0.225	0.063	94.2
MR011111	1.104	0.204	95.5	0.120	0.131	94.4	0.177	0.062	94.3
MR111111	0.800	0.249	96.7	-0.225	0.141	97.2	0.231	0.063	94.5

*Bias (%)* mean relative bias, *RMSE* root mean square error, *CR* coverage rate, *IPW* inverse probability weighting, *OR* outcome regression, *MR* multiply robust, MR: estimators are denoted as “MR000000”, where each digit of the four numbers, from left to right, indicates if ${\pi }^{1}\left({\varvec{X}}\right)$, ${\pi }^{2}\left({\varvec{X}}\right)$,${\pi }^{3}\left({\varvec{X}}\right),{m}^{1}\left({\varvec{X}},Z\right)$, ${m}^{2}\left({\varvec{X}},Z\right)$ or ${m}^{3}\left({\varvec{X}},Z\right)$ is included in the estimator (“1” means yes and “0” means no)

**Table 2:** Simulation results with different sample sizes = 200, 500 or 2000 in the situation where the parametric models did not include the correct models and the neural network model included true covariates

Estimator	*N* = 200			*N* = 500			*N* = 2000		
	Bias (%)	RMSE	CR (%)	Bias (%)	RMSE	CR (%)	Bias (%)	RMSE	CR (%)
IPW.model1	6.356	0.624	98.2	5.464	0.273	99.5	2.539	0.115	97.2
IPW.model2	46.619	0.567	69.6	44.788	0.491	42.6	45.272	0.465	1.2
IPW.model3	67.238	0.708	27.0	66.532	0.680	0.5	67.402	0.677	0
OR.model1	-0.983	0.197	97.6	-1.139	0.112	98.3	0.484	0.054	98.1
OR.model2	42.129	0.484	57.2	40.344	0.431	24.3	41.260	0.420	0.1
OR.model3	67.206	0.714	20.3	66.563	0.683	0.7	67.402	0.678	0
MR100000	6.289	0.372	98.1	4.006	0.210	97.4	1.397	0.100	95.5
MR010000	46.435	0.535	58.5	45.227	0.484	28.0	45.235	0.461	0
MR001000	67.323	0.708	15.9	66.650	0.681	0.4	67.405	0.677	0
MR000100	0.421	0.192	96.2	-0.975	0.112	97.6	0.455	0.055	97.8
MR000010	42.265	0.487	58.3	40.458	0.433	27.0	41.312	0.421	0.1
MR000001	67.395	0.715	19.9	66.618	0.683	0.7	67.425	0.678	0
MR100100	1.217	0.274	97.4	-0.427	0.154	98.0	0.085	0.075	95.8
MR010010	45.952	0.528	57.3	44.782	0.479	25.7	45.093	0.459	0
MR100010	4.864	0.355	98.4	2.818	0.202	97.8	1.105	0.096	95.6
MR010100	1.584	0.212	96.1	-0.283	0.133	95.5	-0.169	0.072	97.1
MR110000	5.109	0.380	98.1	4.001	0.210	97.3	1.501	0.100	95.3
MR000110	0.766	0.193	96.0	-0.648	0.116	96.6	0.328	0.057	97.4
MR000101	0.887	0.194	95.9	-0.722	0.111	97.3	0.498	0.055	97.8
MR000011	41.818	0.484	58.4	40.029	0.429	27.8	40.989	0.417	0.1
MR001100	0.484	0.181	97.8	-0.834	0.106	97.3	0.542	0.054	96.9
MR101000	5.245	0.389	98.5	4.602	0.219	97.9	1.414	0.098	94.6
MR001001	66.695	0.703	18.8	66.293	0.678	0.5	67.372	0.677	0
MR111000	4.091	0.389	98.8	4.438	0.218	97.9	1.583	0.099	94.7
MR000111	1.185	0.195	95.5	-0.481	0.116	96.1	0.426	0.057	97.6
MR001101	1.076	0.275	97.0	-0.302	0.154	98.3	0.172	0.074	96.1
MR110100	1.283	0.279	96.9	-0.446	0.153	98.1	0.075	0.075	95.8
MR100110	0.668	0.277	97.8	-0.484	0.155	97.6	0.159	0.074	95.9
MR101101	0.215	0.269	97.6	-0.354	0.151	98.1	0.242	0.071	95.8
MR110110	0.954	0.274	97.4	-0.483	0.154	98.0	0.165	0.073	96.1
MR011011	44.420	0.512	61.9	43.909	0.467	24.2	44.925	0.457	0
MR111011	5.377	0.356	98.3	4.277	0.203	97.8	1.426	0.091	95.5
MR011111	2.146	0.202	97.5	-0.015	0.123	96.9	0.070	0.063	96.6
MR111111	0.575	0.263	98.2	-0.327	0.151	98.0	0.254	0.070	96.0

*Bias (%)* mean relative bias, *RMSE* Root mean square error, *CR* Coverage rate, *IPW* Inverse probability weighting, *OR* Outcome regression, *MR* Multiply robust; MR estimators are denoted as “MR000000”, where each digit of the four numbers, from left to right, indicates if ${\pi }^{1}\left({\varvec{X}}\right)$, ${\pi }^{2}\left({\varvec{X}}\right)$,${\pi }^{3}\left({\varvec{X}}\right),{m}^{1}\left({\varvec{X}},Z\right)$, ${m}^{2}\left({\varvec{X}},Z\right)$ or ${m}^{3}\left({\varvec{X}},Z\right)$ is included in the estimator (“1” means yes and “0” means no)

According to Table 1 and Figure S1, biases of IPW, OR, or MR estimators were ignorable when the parametric models were correctly specified or NNET models were included. The RMSEs of estimators with correct PS or NN-PS models were larger than those with correct OR or NN-OR, respectively. And the RMSEs of the estimators with the correct parametric OR model (OR.model2, MR000010) had the smallest RMSEs among all estimators, and were significantly less than those with NN-OR model (OR.model1, MR000100). However, it can easily be seen that the biases and RMSEs of the estimator with a wrong parametric model are much larger (OR.model3, MR000001).

Together with Table 2 and Figure S2, the proposed MR estimators improved the robustness to the model misspecification even if the parametric models were all incorrectly modeled (MR111000, MR000111, MR111011, MR011111, MR111111 in Table 2 and Figure S2). Although the biases of the six estimators are negligible, the MR000111 had the smallest RMSE among the six estimators. Further, MR000111 with a correct OR model (MR000111 in Table 1 and Figure S1) had a smaller RMSE than the MR estimators with both parametric PS and OR models; and the RMSE of MR000111 was small as that of OR estimator with the correct parametric model (OR.model2 in Table 1 and Figure S1). Even if the two parametric OR models are incorrectly specified, the RMSE of MR000111 is similar to that of NN-OR model.

In addition, Table 3 and Figure S3 showed the simulation results when NNET models included all covariates where parametric models included a correct model; and Table 4 and Figure S4 showed the simulation results when NNET models included all covariates where parametric models did not include a correct model. Similar results were observed in Tables 3 and 4 and Figures S3 and S4.

**Table 3:** Simulation results with different sample sizes = 200, 500 or 2000 in the situation where the parametric models included the correct models and the neural network model included all covariates

Estimator	*N* = 200			*N* = 500			*N* = 2000		
	Bias (%)	RMSE	CR (%)	Bias (%)	RMSE	CR (%)	Bias (%)	RMSE	CR (%)
IPW.model1	4.087	0.756	99.3	4.534	0.327	99.6	2.474	0.116	98.2
IPW.model2	7.234	0.472	98.8	1.497	0.306	96.2	0.702	0.166	95.5
IPW.model3	67.238	0.708	27.0	66.532	0.680	0.5	67.402	0.677	0
OR.model1	-3.473	0.236	97.7	-1.343	0.130	98.6	0.430	0.059	98.7
OR.model2	0.713	0.157	94.4	-0.544	0.095	95.1	0.245	0.046	95.6
OR.model3	67.206	0.714	20.3	66.563	0.683	0.7	67.402	0.678	0
MR100000	5.769	0.393	98.1	4.767	0.220	98.6	1.275	0.092	96.1
MR010000	3.434	0.292	93.7	1.196	0.194	92.3	0.604	0.100	93.4
MR001000	67.323	0.708	15.9	66.650	0.681	0.4	67.405	0.677	0
MR000100	1.296	0.221	97.5	-0.800	0.126	98.4	0.383	0.060	98.5
MR000010	0.737	0.159	93.7	-0.441	0.097	95.2	0.253	0.046	95.3
MR000001	67.395	0.715	19.9	66.618	0.683	0.7	67.425	0.678	0
MR100100	2.534	0.288	98.2	-0.128	0.164	98.4	0.135	0.075	97.4
MR010010	1.053	0.197	94.3	-0.201	0.127	93.2	0.131	0.061	93.7
MR100010	1.737	0.250	97.9	-0.037	0.142	97.7	0.156	0.063	94.6
MR010100	1.401	0.242	95.7	-0.059	0.149	96.2	-0.101	0.074	96.9
MR110000	4.103	0.398	98.8	1.673	0.210	98.2	1.090	0.091	96.1
MR000110	-3.647	0.178	96.7	-2.681	0.100	96.9	-0.272	0.046	96.7
MR000101	1.986	0.219	97.3	-0.578	0.126	98.3	0.440	0.060	98.5
MR000011	1.155	0.159	94.2	-0.212	0.098	94.9	0.307	0.046	95.5
MR001100	1.435	0.205	97.6	-0.645	0.121	98.0	0.408	0.058	98.4
MR101000	4.584	0.403	98.8	5.113	0.211	98.7	1.261	0.089	97.0
MR001001	66.695	0.703	18.8	66.293	0.678	0.5	67.372	0.677	0
MR111000	2.864	0.398	99.1	2.326	0.205	98.6	1.060	0.088	96.7
MR000111	-3.181	0.177	96.9	-2.429	0.101	97.2	-0.215	0.046	96.8
MR001101	3.140	0.291	98.3	0.087	0.162	98.8	0.137	0.074	97.3
MR110100	2.774	0.288	97.8	-0.107	0.164	98.2	0.112	0.075	97.1
MR100110	2.179	0.257	97.1	-0.374	0.141	97.8	0.235	0.063	95.8
MR101101	2.972	0.293	98.3	0.054	0.159	99.0	0.087	0.072	97.6
MR110110	2.384	0.262	97.8	-0.257	0.143	98.0	0.207	0.064	95.7
MR011011	1.383	0.204	94.5	0.060	0.131	94.5	0.209	0.062	94.2
MR111011	1.983	0.275	97.7	0.168	0.148	97.7	0.120	0.064	94.9
MR011111	1.017	0.203	95.6	0.011	0.130	95.2	0.254	0.061	95.4
MR111111	2.366	0.268	97.5	-0.039	0.149	98.0	0.177	0.064	95.6

*Bias (%)* mean relative bias, *RMSE* root mean square error, *CR* coverage rate, *IPW* inverse probability weighting, *OR* outcome regression, *MR* multiply robust, MR estimators are denoted as “MR000000”, where each digit of the four numbers, from left to right, indicates if ${\pi }^{1}\left({\varvec{X}}\right)$, ${\pi }^{2}\left({\varvec{X}}\right)$,${\pi }^{3}\left({\varvec{X}}\right),{m}^{1}\left({\varvec{X}},Z\right)$, ${m}^{2}\left({\varvec{X}},Z\right)$ or ${m}^{3}\left({\varvec{X}},Z\right)$ is included in the estimator (“1” means yes and “0” means no)

**Table 4:** Simulation results with different sample sizes = 200, 500 or 2000 in the situation where the parametric models did not include the correct models and the neural network model included all covariates

Estimator	*N* = 200			*N* = 500			*N* = 2000		
	Bias (%)	RMSE	CR (%)	Bias (%)	RMSE	CR (%)	Bias (%)	RMSE	CR (%)
IPW.model1	4.087	0.756	99.3	4.534	0.327	99.6	2.474	0.116	98.2
IPW.model2	46.619	0.567	69.6	44.788	0.491	42.6	45.272	0.465	1.2
IPW.model3	67.238	0.708	27.0	66.532	0.680	0.5	67.402	0.677	0
OR.model1	-3.473	0.236	97.7	-1.343	0.130	98.6	0.430	0.059	98.7
OR.model2	42.129	0.484	57.2	40.344	0.431	24.3	41.260	0.420	0.1
OR.model3	67.206	0.714	20.3	66.563	0.683	0.7	67.402	0.678	0
MR100000	5.769	0.393	98.1	4.767	0.220	98.6	1.275	0.092	96.1
MR010000	46.435	0.535	58.5	45.227	0.484	28.0	45.235	0.461	0
MR001000	67.323	0.708	15.9	66.650	0.681	0.4	67.405	0.677	0
MR000100	1.296	0.221	97.5	-0.800	0.126	98.4	0.383	0.060	98.5
MR000010	42.265	0.487	58.3	40.458	0.433	27	41.312	0.421	0.1
MR000001	67.395	0.715	19.9	66.618	0.683	0.7	67.425	0.678	0
MR100100	2.534	0.288	98.2	-0.128	0.164	98.4	0.135	0.075	97.4
MR010010	45.952	0.528	57.3	44.782	0.479	25.7	45.093	0.459	0
MR100010	4.220	0.397	98.4	3.676	0.215	98.7	0.926	0.089	96.8
MR010100	1.865	0.229	96.5	-0.101	0.141	97.4	-0.359	0.075	97.2
MR110000	4.939	0.404	98.6	5.023	0.219	98.6	1.464	0.092	96.2
MR000110	1.280	0.223	96.6	-0.359	0.130	97.6	0.218	0.063	98.1
MR000101	1.986	0.219	97.3	-0.578	0.126	98.3	0.440	0.060	98.5
MR000011	41.818	0.484	58.4	40.029	0.429	27.8	40.989	0.417	0.1
MR001100	1.435	0.205	97.6	-0.645	0.121	98.0	0.408	0.058	98.4
MR101000	4.584	0.403	98.8	5.113	0.211	98.7	1.261	0.089	97.0
MR001001	66.695	0.703	18.8	66.293	0.678	0.5	67.372	0.677	0
MR111000	3.688	0.408	98.8	5.317	0.214	98.7	1.400	0.089	96.3
MR000111	1.888	0.221	96.9	-0.163	0.128	97.6	0.293	0.062	98.0
MR001101	3.140	0.291	98.3	0.087	0.162	98.8	0.137	0.074	97.3
MR110100	2.706	0.293	98.3	-0.023	0.165	98.6	0.097	0.076	97.6
MR100110	2.341	0.290	97.9	0.091	0.163	98.5	0.149	0.073	97.6
MR101101	2.972	0.293	98.3	0.054	0.159	99.0	0.087	0.072	97.6
MR110110	2.789	0.292	97.9	0.116	0.164	98.5	0.130	0.074	97.4
MR011011	44.420	0.512	61.9	43.909	0.467	24.2	44.925	0.457	0
MR111011	5.161	0.395	98.7	5.598	0.208	98.8	1.538	0.087	96.4
MR011111	2.330	0.218	97.6	0.091	0.130	98.1	0.055	0.067	97.4
MR111111	2.725	0.289	98.3	0.402	0.157	99.0	0.107	0.071	97.3

*Bias (%)* mean relative bias, *RMSE* Root mean square error, *CR* Coverage rate, *IPW*: inverse probability weighting; OR: outcome regression; MR, multiply robust; MR estimators are denoted as “MR000000”, where each digit of the four numbers, from left to right, indicates if ${\pi }^{1}\left({\varvec{X}}\right)$, ${\pi }^{2}\left({\varvec{X}}\right)$,${\pi }^{3}\left({\varvec{X}}\right),{m}^{1}\left({\varvec{X}},Z\right)$, ${m}^{2}\left({\varvec{X}},Z\right)$ or ${m}^{3}\left({\varvec{X}},Z\right)$ is included in the estimator (“1” means yes and “0” means no)

In conclusion, the simulation results showed that the proposed MR estimators were robust to model misspecification even if all parametric models were incorrectly specified. Further, considering the robustness to model misspecification and RMSE, the MR estimators with only OR models, where one of the models was a NNET model, was the most recommended. The recommended estimators were robust to model misspecification and tended to have the smallest RMSE when the estimators included a correct OR model; and the performance of the recommended estimators was comparative even if all parametric models were misspecified.

### Empirical study

The China Health and Retirement Longitudinal Study (CHARLS) is a large-scale, nationally representative longitudinal survey of people aged 45 or older and their spouses in China, including assessments of the social, economic, and health status of community residents [25]. The study aimed to estimate the treatment effect of social activity on the depression level in the real-world data from CHARLS. The depression level was evaluated by CES-D Depression Scale, and the total score was between 0 and 30: a higher total score denotes a higher depression level, while a lower total score denotes a lower depression level. The self-reported social activity includes 11 categories based on individual responses to the question, “Have you done any of these activities in the last month”. The value of the variable is 1 if the participant takes part in any activities; otherwise, the value is 0. The group with a value 1 was defined as a social activity group, and the group with a value 0 was defined as a non-social activity group. Baseline information included age, marital status, sex, region, smoke status, self-reported hypertension, self-reported diabetes, self-reported heart disease, and self-reported stroke. Inclusion criteria are: (1) participants who took part in the survey in 2011–2012 (2) complete baseline. A total of 10,119 participants were included in the analysis. The baseline information was summarized in Table S1. We found that the non-social activity group had a higher level of depression.

In the study, we specified three PS models (including one NN-PS model and two logistic models) and three OR models (including one NN-OR model and two linear regression) in the MR method. For the NNET models, we included all covariates. For the parametric models, we explored the association of social activity group/non-social activity group with all covariates via a logistic model, and the association of depression with all covariates through a linear model; and we identified candidate models with a significant level at 0.05 and 0.01. Hence, three set of covariates in [${\pi }^{1}\left({\varvec{X}}\right),{\pi }^{2}\left({\varvec{X}}\right),{\pi }^{3}({\varvec{X}})$] are: (i) age, marital status, sex, region, smoke status, self-reported hypertension, self-reported diabetes, self-reported heart disease and self-reported stroke; (ii) age, marital status, sex, region, smoke status, and self-reported diabetes; (iii) age, region and smoke status. Three sets of covariates in [${m}^{1}({\varvec{X}},Z),{m}^{2}({\varvec{X}},Z),{m}^{3}({\varvec{X}},Z)$] are: (i) age, marital status, sex, region, smoke status, self-reported hypertension, self-reported diabetes, self-reported heart disease, self-reported stroke and activity group; (ii) marital status, sex, region, self-reported diabetes, self-reported heart disease, self-reported stroke, and social activity; (iii) marital status, sex, region, self-reported heart disease, self-reported stroke and activity group. We applied the MR methods to estimate the effect. The results were shown in Table 5 and Figure S5.

**Table 5:** Estimating effect of social activity on depression level (non-social activity group as reference group)

Estimator	Estimate	95%CI	BS-SE
MR100000	-0.642	(-0.861, -0.423)	0.112
MR010000	-0.525	(-0.715, -0.335)	0.097
MR001000	-0.493	(-0.684, -0.302)	0.098
MR000100	-0.526	(-0.722, -0.330)	0.100
MR000010	-0.492	(-0.682, -0.303)	0.097
MR000001	-0.488	(-0.678, -0.298)	0.097
MR100100	-0.526	(-0.751, -0.301)	0.115
MR010010	-0.507	(-0.697, -0.317)	0.097
MR100010	-0.492	(-0.682, -0.303)	0.097
MR010100	-0.521	(-0.712, -0.331)	0.097
MR110000	-0.525	(-0.715, -0.335)	0.097
MR000110	-0.504	(-0.693, -0.315)	0.097
MR000101	-0.503	(-0.693, -0.313)	0.097
MR000011	-0.493	(-0.682, -0.303)	0.097
MR001100	-0.522	(-0.715, -0.330)	0.098
MR101000	-0.493	(-0.683, -0.303)	0.097
MR001001	-0.502	(-0.692, -0.311)	0.097
MR111000	-0.525	(-0.716, -0.334)	0.097
MR000111	-0.504	(-0.693, -0.315)	0.096
MR001101	-0.503	(-0.693, -0.314)	0.097
MR110100	-0.521	(-0.711, -0.331)	0.097
MR100110	-0.504	(-0.694, -0.315)	0.097
MR101101	-0.510	(-0.700, -0.320)	0.097
MR110110	-0.511	(-0.702, -0.321)	0.097
MR011011	-0.507	(-0.697, -0.317)	0.097
MR111011	-0.507	(-0.697, -0.317)	0.097
MR011111	-0.511	(-0.702, -0.321)	0.097
MR111111	-0.511	(-0.702, -0.321)	0.097

*BS-SE* Bootstrap standard error based on 200 resamples, *95% CI* 95% confidence interval

From Table 5, all estimates showed that the social activity group had lower depression scores than the non-social activity group. However, when only specifying NN-PS model and NN-OR model (MR100000, MR000100, MR100100), the estimators tended to have higher estimated values and higher standard errors. The other estimators had similar estimates, and MR000111 tended to have the smallest standard errors.

## Discussion

In this study, we considered estimating ATE between treatment and control groups in observational studies. The proposed MR estimators combined parametric and nonparametric models based on the previous MR method [8]. Our simulation study showed that the MR estimators with only outcome regression (OR) models, where one of the models was a nonparametric model, were the most recommended because of the robustness to model misspecification and the lowest root mean square error (RMSE) when including a correct parametric OR model. And the performance of the recommended estimators was comparative even if all parametric models were misspecified. We mainly focused on estimating ATE in the study, and our proposed method can be easily to estimate other causal parameters, such as the average treatment effect on the treated, $E({Y}^{1}-{Y}^{0}|Z=1)$ [26], and log of causal risk ratio for binary outcome $log\frac{E({Y}^{1})}{E({Y}^{0})}$ [27].

Our simulation results showed that the IPW, direct confounding adjustment, or MR estimators with only parametric models might gain a large bias, large RMSE, and low coverage rate when the parametric models were misspecified. By contrast, when adding a NNET model to the MR estimators, the bias was ignorable and coverage rate was close to 95% even if we misspecified all parametric models. In addition, the estimators with only OR models are the most recommended because of the robustness to the model specification and the smallest RMSE when including a correct OR model. Further, NNET can extract the complex relationship among variables without prior information so that we put some variables unrelated to exposure or outcome in the model and could still get similar results.

A limitation of study is that no relevant theoretical proof was provided, and future research will focus on theoretical proofs and properties of the proposed method. Further, we focused on estimating ATE in a non-survival context in the study, but there are lots of time-to-event outcomes in observational studies, and the extension of the proposed method in the survival outcomes studies will be a topic of our future research.

## Conclusions

In this study, we proposed a new MR estimator, considering nonparametric and parametric models, which is more robust to model misspecification.

### Supplementary Information

**Additional file 1.**

## Data Availability

Publicly available datasets were analyzed in this study. These data can be downloaded from http://charls.pku.edu.cn/.

## Abbreviations

MR: Multiply robust
ATE: Average treatment effect
RMSE: Root mean square error
IPW: Inverse probability weighting
PS: Propensity score
OR: Outcome regression
NNET: Neural network
CHARLS: China Health and Retirement Longitudinal Study
