# Supplementary material for: An improved multiply robust estimator for the average treatment effect
Source: BMC Med Res Methodol. 2023 Oct 11;23:231. doi: 10.1186/s12874-023-02056-7 (PMC10568861; doi:10.1186/s12874-023-02056-7)
Supplement: Supplementary file 1 — Additional file 1. [file 12874_2023_2056_MOESM1_ESM.pdf]

Supplementary materials for "An improved multiply robust estimator for the average treatment effect"

Ce Wang*, Kecheng Wei*, Chen Huang, Yongfu Yu, Guoyou Qin

Department of Biostatistics, Key Laboratory for Health Technology Assessment, National Commission of Health, Key Laboratory of Public Health Safety of Ministry of Education, School of Public Health, Fudan University, Shanghai, China

* These authors contributed equally.

Correspondence to: Guoyou Qin gyqin@fudan.edu.cn,Yongfu Yu yu@fudan.edu.cn

Table S1. Baseline characteristics between chemotherapy and non-chemotherapy groups

	Non-social activity group	Social activity group	
Marital			<0.001
Yes	3831 (77.5)	4203 (81.2)	
No	1111 (22.5)	974 (18.8)	
Sex			0.083
Male	2484 (50.3)	2512 (48.5)	
Female	2458 (49.7)	2665 (51.5)	
Urban			<0.001
Yes	960 (19.4)	1502 (29.0)	
No	3982 (80.6)	3675 (71.0)	
Smoke			0.728
Yes	2006 (40.6)	2120 (41.0)	
No	2936 (59.4)	3057 (59.0)	
Hypertension			0.185
Yes	1262 (25.5)	1383 (26.7)	
No	3680 (74.5)	3794 (73.3)	
Diabetes			0.002
Yes	255 (5.2)	343 (6.6)	
No	4687 (94.8)	4834 (93.4)	
Heart disease			0.619
Yes	659 (13.3)	672 (13.0)	
No	4283 (86.7)	4505 (87.0)	
Stroke			0.776
Yes	112 (2.3)	112 (2.2)	
No	4830 (97.7)	5065 (97.8)	
Depression level (mean±SD)	20.42 (5.22)	19.78 (4.72)	<0.001
Age (mean±SD)	60.74 (10.19)	58.99 (10.14)	<0.001

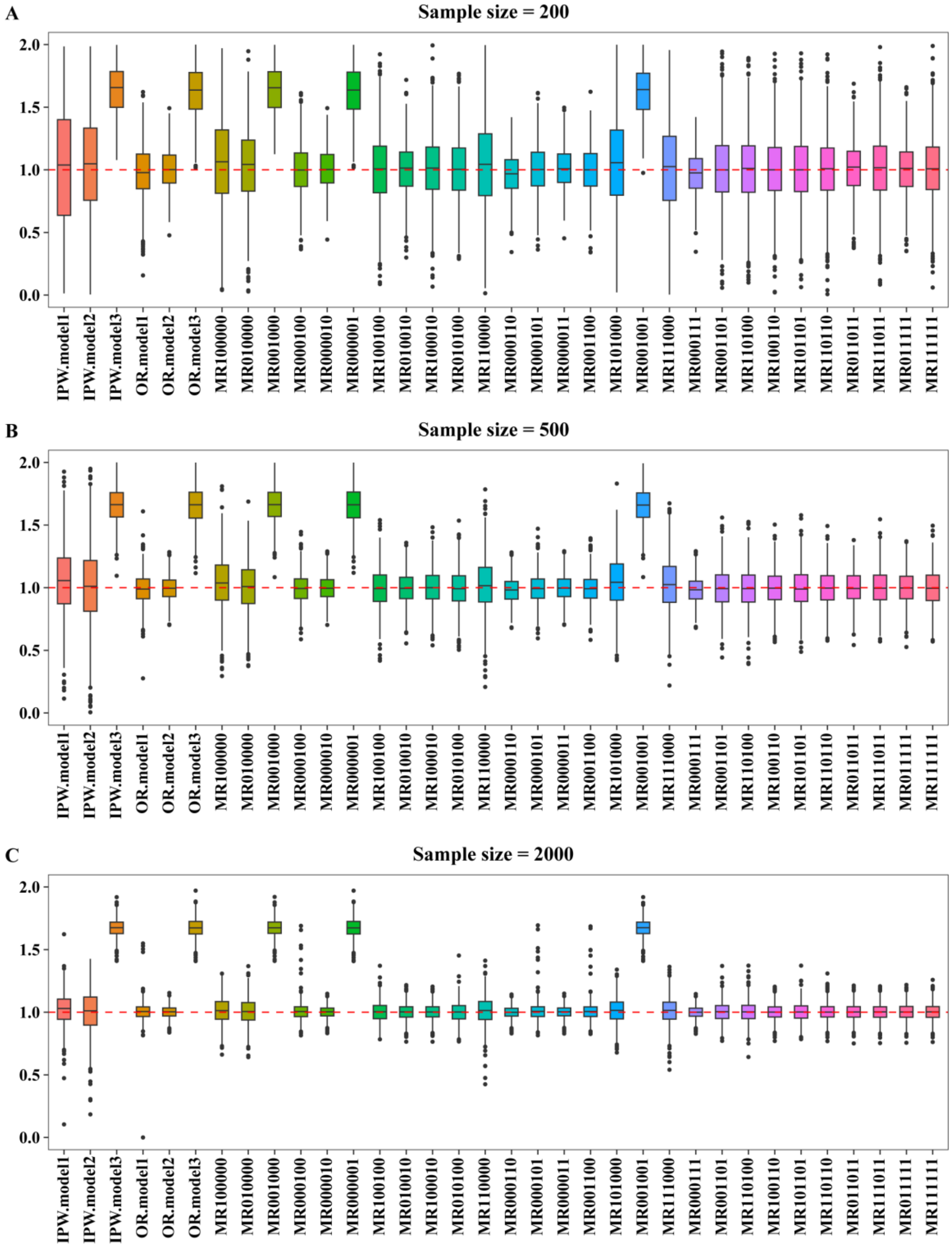

Figure S1. Simulation results with different sample sizes = 200, 500 or 2000 in the situation where the parametric models included the correct models and the neural network model included true covariates.

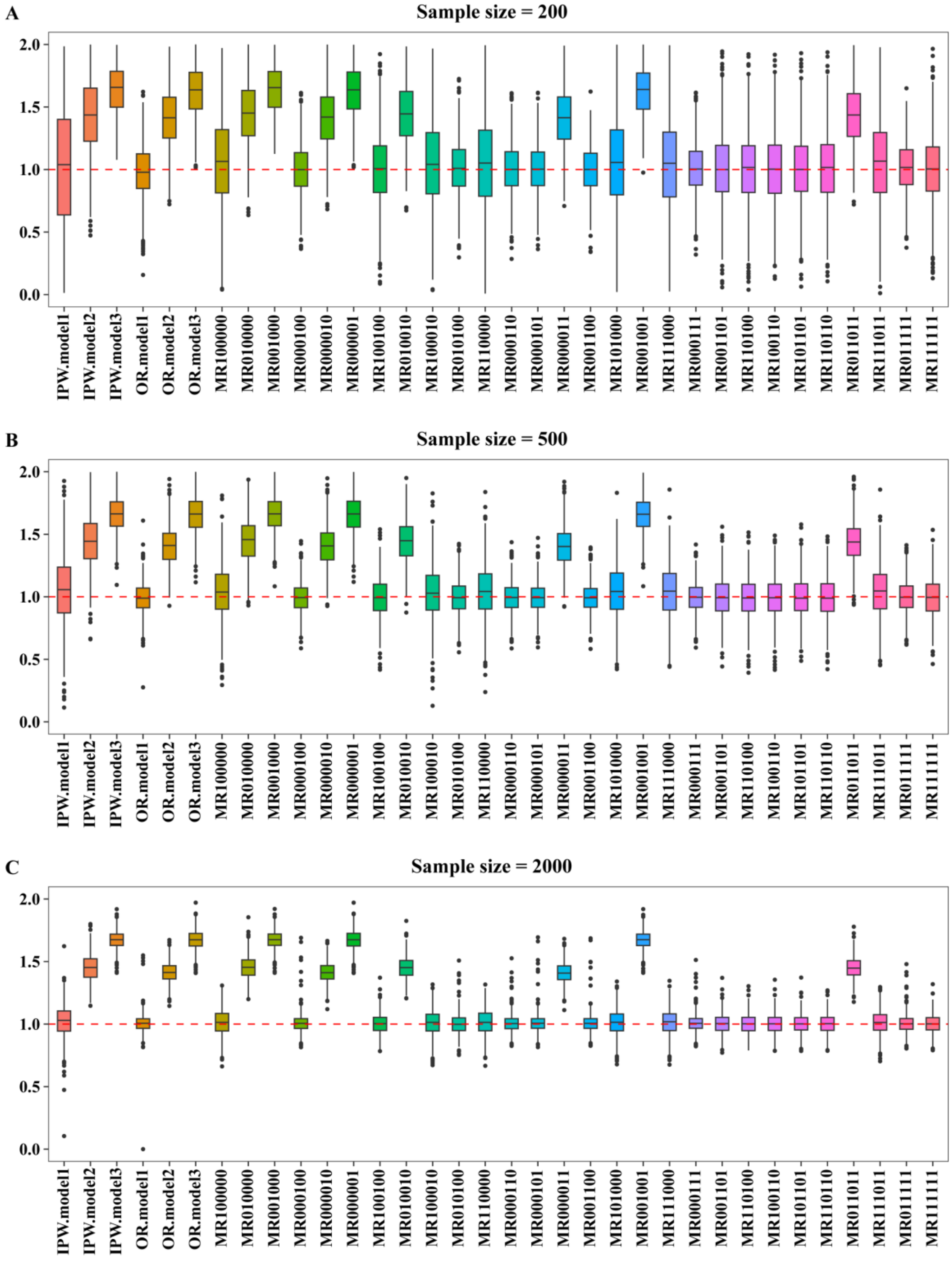

Figure S2. Simulation results with different sample sizes = 200, 500 or 2000 in the situation where the parametric models did not include the correct models and the neural network model included true covariates.

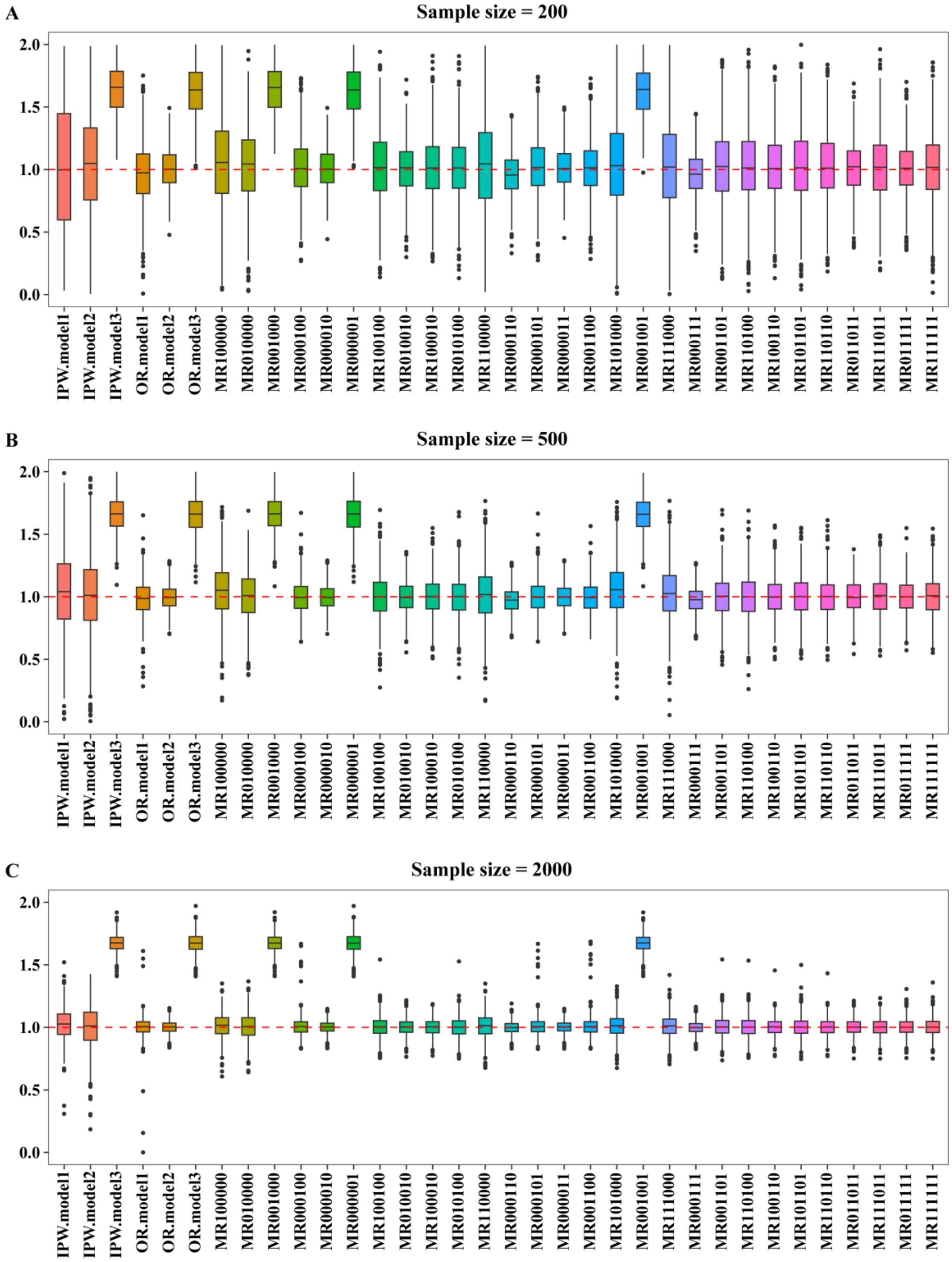

Figure S3. Simulation results with different sample sizes = 200, 500 or 2000 in the situation where the parametric models included the correct models and the neural network model included all covariates.

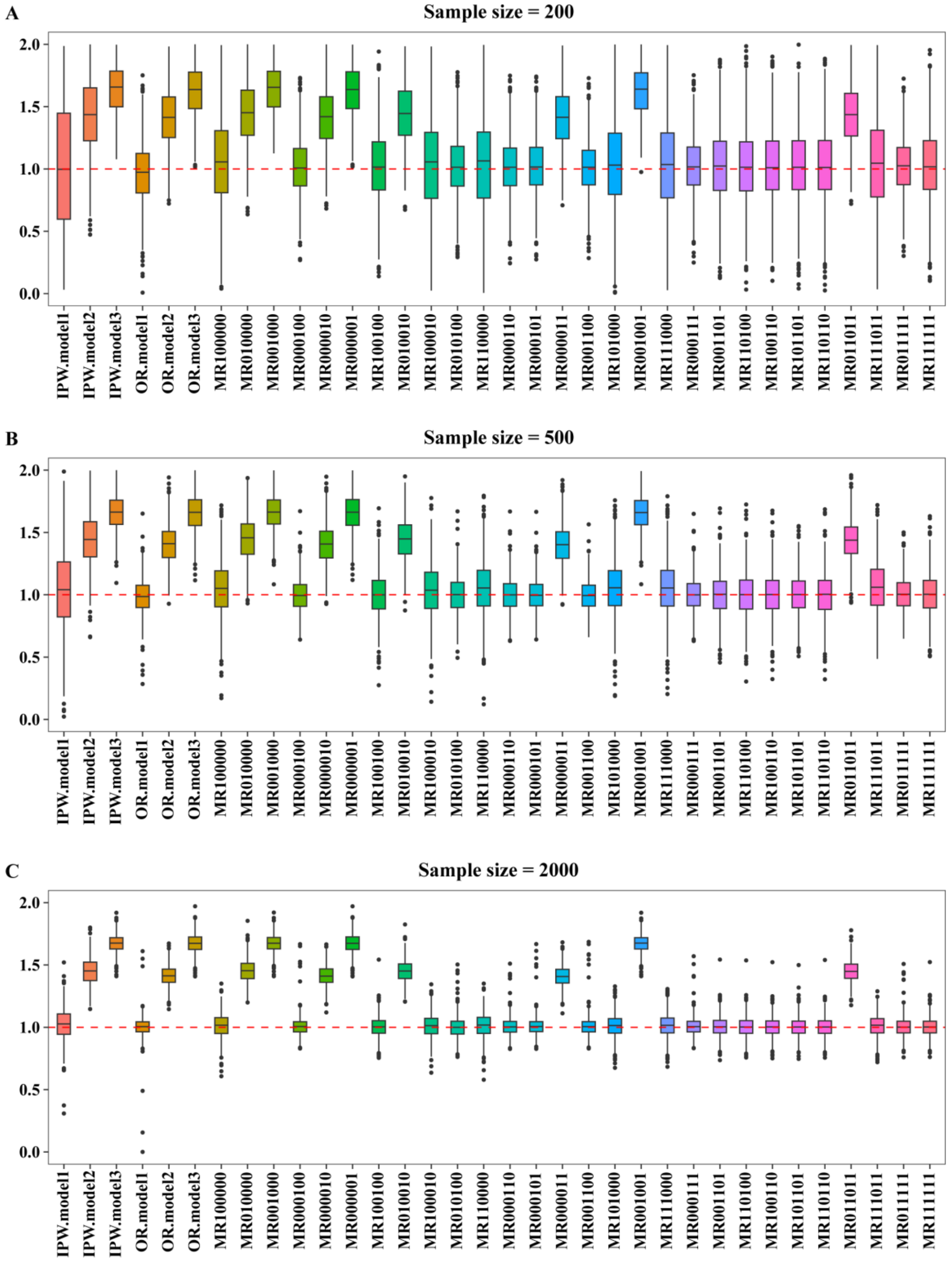

Figure S4. Simulation results with different sample sizes = 200, 500 or 2000 in the situation where the parametric models did not include the correct models and the neural network model included all covariates
